# An *In Vivo* Mouse Model of Pelvic Recurrence of Human Colorectal Cancer

**DOI:** 10.1038/s41598-019-56152-0

**Published:** 2019-12-23

**Authors:** Masashi Yamamoto, Kohei Taniguchi, Shinsuke Masubuchi, Tomo Tominaga, Yosuke Inomata, Akiko Miyamoto, Taka-Aki Ishizuka, Takashi Murakami, Wataru Osumi, Hiroki Hamamoto, Keitaro Tanaka, Junji Okuda, Kazuhisa Uchiyama

**Affiliations:** 1 Departments of General and Gastroenterological Surgery, Osaka Medical College, Osaka, Japan; 2 Translational Research Program, Osaka Medical College, Osaka, Japan; 3 Division of Research Equipment and Device, Osaka Medical College, Osaka, Japan; 4 Faculty of Medicine, Saitama Medical University, Saitama, Japan

**Keywords:** Cancer imaging, Cancer models

## Abstract

Pelvic recurrence of colorectal cancer is a crucial problem because radical surgery can lead to excessive invasion. Novel therapeutic strategies are required instead of surgery. However, there are few suitable models because of the difficulty in transplanting and observing tumors in the pelvis. We have established an appropriate injection site suitable for the establishment of colorectal cancer pelvic recurrence that allows for the observation of tumor growth. DLD-1 cells stably expressing luciferase (DLD-1 clone#1-Luc) were inoculated into various points of female BALB/c nude mice and the engrafted cells were analyzed with an imaging system employing bioluminescent signals and computed tomography. Weekly analysis with the imaging system showed that a triangular area defined by the vagina, the anus, and the ischial spine was suitable for the engraftment of pelvic tumors. The imaging system was able to detect the engrafted tumor 7 days after the inoculation of cells. Weight loss was observed in our model, and overall survival was 21–42 days. Tumor involvement of adjacent organs was detected histopathologically, as is the case in the clinical situation. These findings suggest that this model is valid for evaluations of the therapeutic effects of novel treatments under development. It is hoped that this model will be used in preclinical research.

## Introduction

Colorectal cancer (CRC) is the third most common cancer worldwide with an estimated 1.4 million new cases per year^1^. Although the introduction of total mesorectal excision and neoadjuvant therapy has improved the prognosis of rectal cancer, local recurrence still occurs in 5% to 13% of patients after curative resection^2–6^. To improve symptom control and long-term survival, R0 resection (no cancer cells seen microscopically at the resection margin) is one of the important prognostic factors for the local recurrence of rectal cancer^7^. To secure the surgical margin, other adjacent organs (i.e., the colon, small intestine, urinary organs and, reproductive organs) are often resected, but radical surgery is highly invasive and it deteriorates quality of life (QOL). Hence, the use of alternative therapies to avoid invasive surgery such as additional (neo) adjuvant chemotherapy, molecular targeting therapy, radiotherapy, and carbon-ion radiation therapy have been investigated^8–11^, and the prompt establishment of a promising alternative therapeutic strategy is eagerly awaited^12^.

In order to examine the effects of novel treatments *in vivo*, an appropriate animal model is essential. However, to the best of our knowledge, there is no suitable pelvic recurrence model of CRC. One of the reasons for the present situation is that the observation of the pelvis in animal models is more difficult compared to the subcutaneous transplant model. However, in recent years, imaging tools for medicine and biomedical research such as ultrasound, magnetic resonance imaging (MRI), computed tomography (CT), and positron emission tomography (PET) have been rapidly developed. Especially in animal experimental models, biomedical imaging systems with luciferase are very useful^13^. In fact, several mouse models of metastatic human cancer labelled with luciferase have been reported^14–19^. Here, we sought to establish an appropriate animal model for the pelvic recurrence of CRC employing the bioluminescent imaging of tumors. The precision and features of our CRC pelvic recurrence model were examined from various viewpoints by imaging and histological assessments.

## Results

### Injection site defined by the triangle of the vagina, anus, and ischial spine

**Figure 1:**
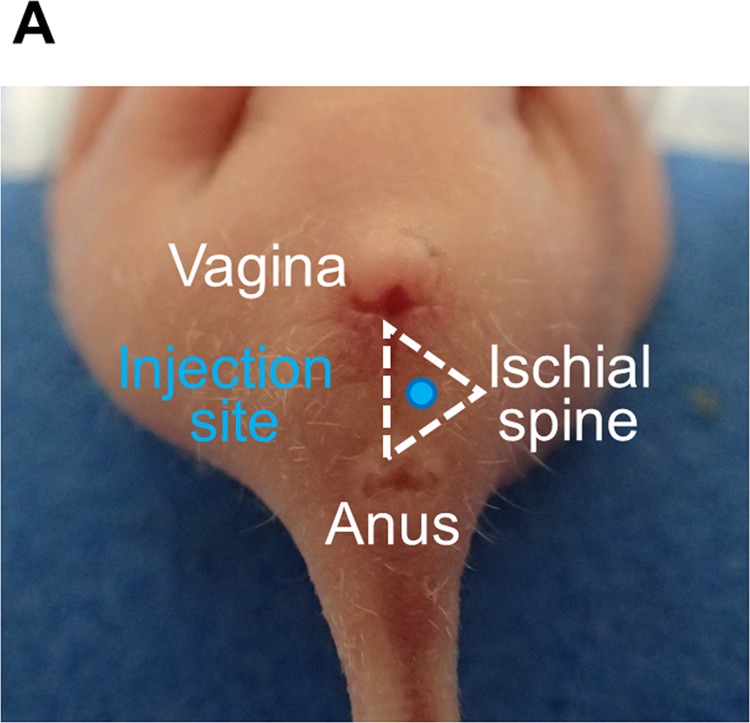
Injection site. The injection site was located inside the triangle defined by the vagina, anus, and ischial spine. DLD-1 clone#1-Luc cells were inoculated horizontally into the injection site with a 27 G needle under anesthesia.

First, we established an appropriate site for our CRC pelvic recurrence model. The injection site was selected within the triangle defined by the vagina, anus, and ischial spine through the consideration of pelvic anatomy (Fig. 1).

### Injected CRC cells grew in the pelvis as in the clinical situation

**Figure 2:**
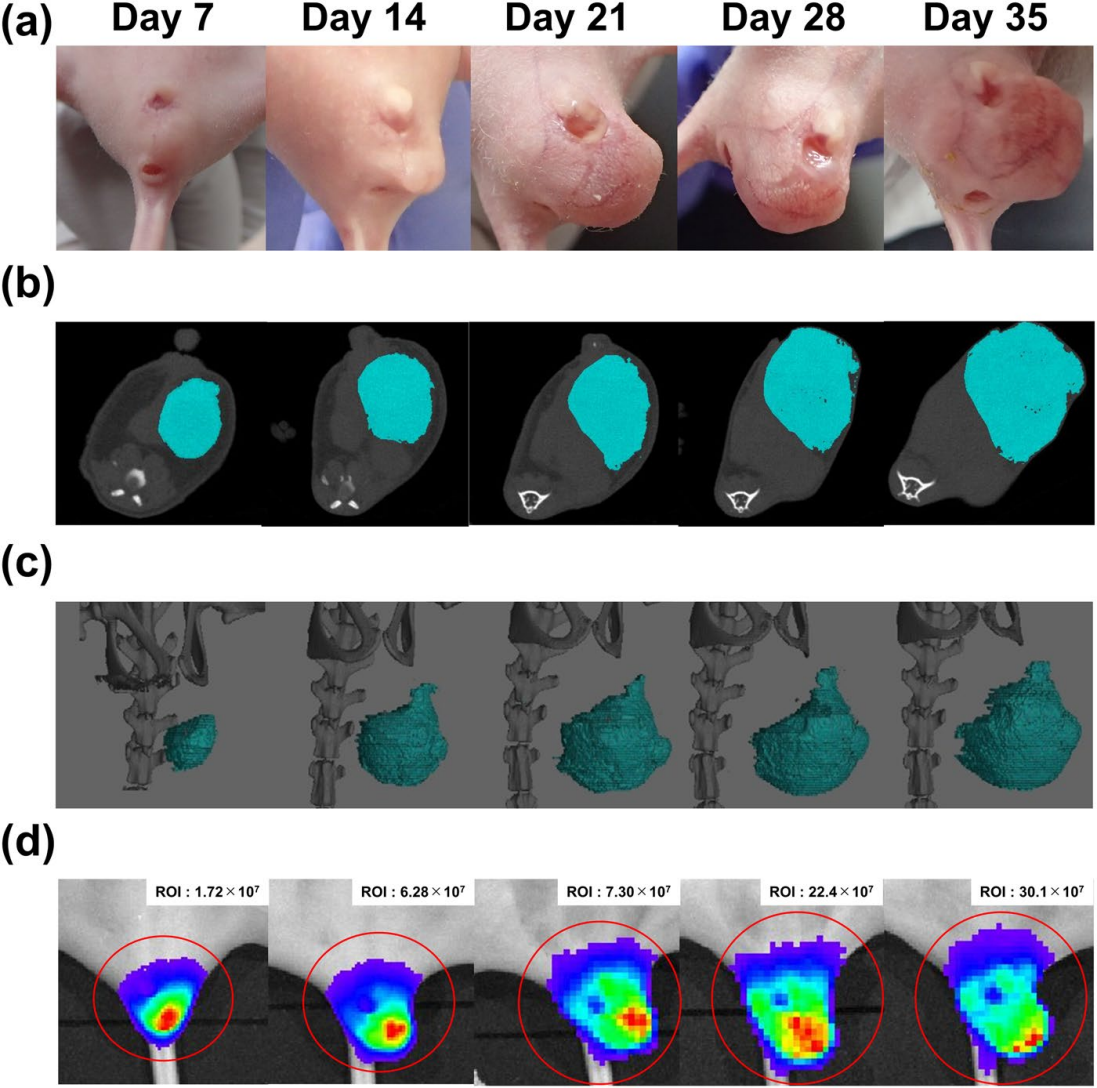
Representative images. (**a**) Images of tumors from the body surface. Images were taken every 7 days. (**b**) Images of tumors using the ROI contour tool with the Latheta CT scanner. (**c**) 3D images of tumors using VG Studio max 2.2. (**d**) Images from the Xenogen IVIS imaging system. (**e**) Total tumor volumes at 7, 14, 21, 28, 35, and 42 days after the inoculation of DLD-1 clone#1-Luc cells. (**f**) Total photon flux results within 6 min of the luciferin injection.

On day 7 after injection, we investigated whether tumors had been transplanted into the pelvis. As shown in Fig. 2a–c, CT scanning revealed engrafted tumors in the pelvis before detection by observation of the surface of the body. The same tendency was observed by assessing the photons emitted from the tumors using the Xenogen IVIS imaging system (Fig. 2d). Two weeks after injection, the developed tumor was discernible, even by naked-eye observation (Fig. 2a). Gradual tumor growth was demonstrated during the observation period by all test methods (Fig. 2a–d). The same injection method also led to the establishment of engrafted tumors in male mice (Supplementary Fig. S1), but tumors were difficult to identify using the CT system due to the narrow pelvic space. Also, mouse-derived Colon26-Luc cells were transplanted into the pelvis of wild type-BALB/c mice (Supplementary Fig. S2). These results suggest that this injection site can be used to establish a mouse model of CRC pelvic recurrence.

### Changes in tumor size, photon flux result, body weight, and survival rate in the CRC pelvic recurrence model

**Figure 3:**
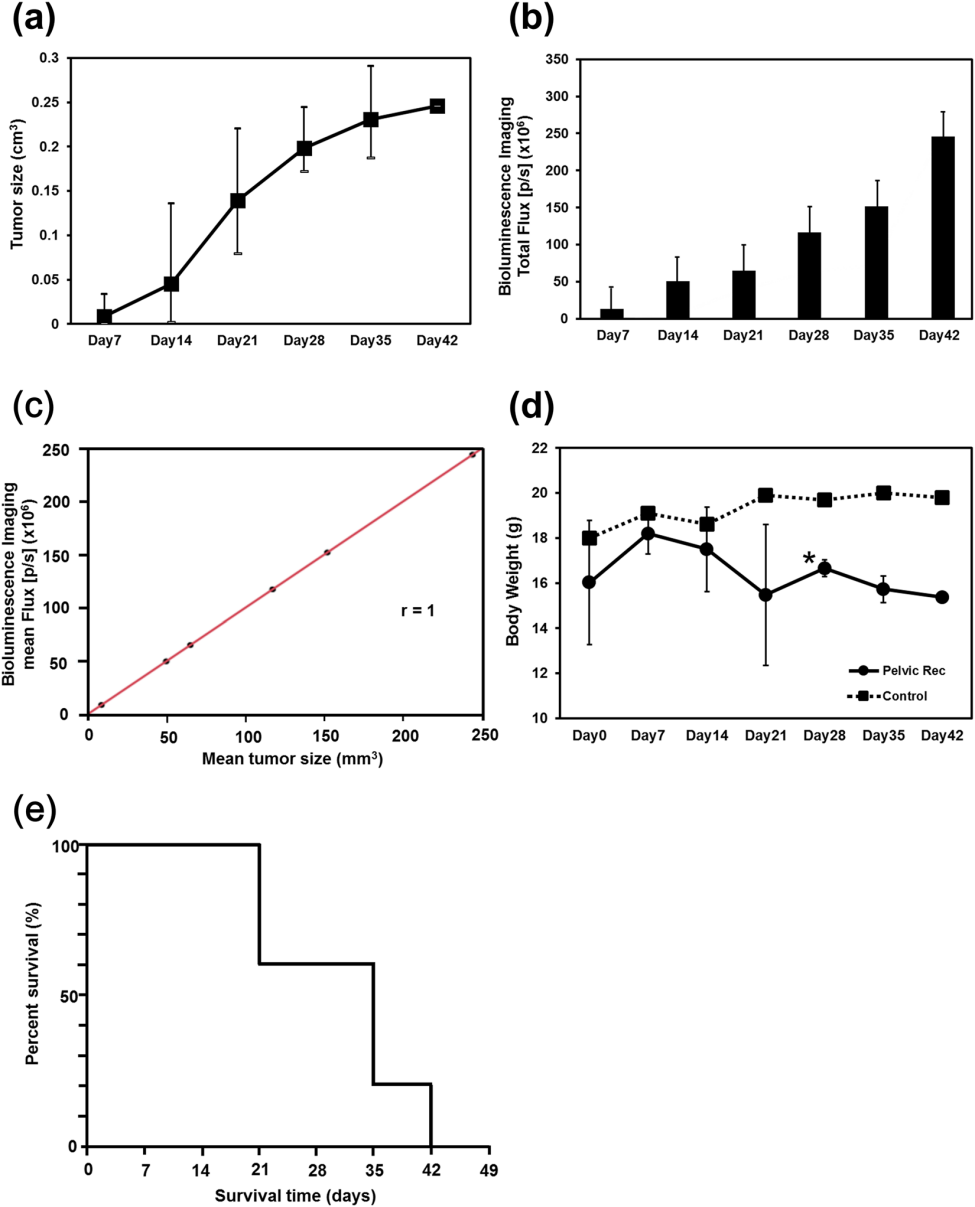
Tumor volume, photon flux result, body weight and overall survival in the CRC pelvic recurrence model. (**a**) Total tumor volumes at 7, 14, 21, 28, 35, and 42 days after the inoculation of DLD-1 clone#1-Luc cells. (**b**) Total photon flux results. (**c**) Analysis of the correlation between tumor volume and photon count; Pearson’s correlation r = 1.0. (**e**) Differences in body weight between control mice and mice in the CRC pelvic recurrence model. Results are presented as the mean ± Standard Deviation (SD); *p < 0.05 (**f**) Kaplan-Meier plot of overall survival of pelvic recurrence model (n = 5).

Tumor volume steadily increased over time (Fig. 3a) and the intensity of luminescent signaling had a positive correlation with increasing tumor volume (Fig. 3b; Pearson correlation r = 0.95, Fig. 3c). As shown in Fig. 3d, body weight (BW) was recorded every 7 days; the results show statistically significant weight loss after 28 days compared to control mice (*p* < 0.02). Regarding the survival time of mice in our CRC pelvic recurrence model, we observed mortality within 21–42 days after the injection of CRC cells (Fig. 3e). These findings suggest that our pelvic recurrence model is useful for the evaluation of therapeutic effects by evaluating BW and survival time.

### Histopathological changes and immunohistochemical analysis reflect the clinical situation

**Figure 4:**
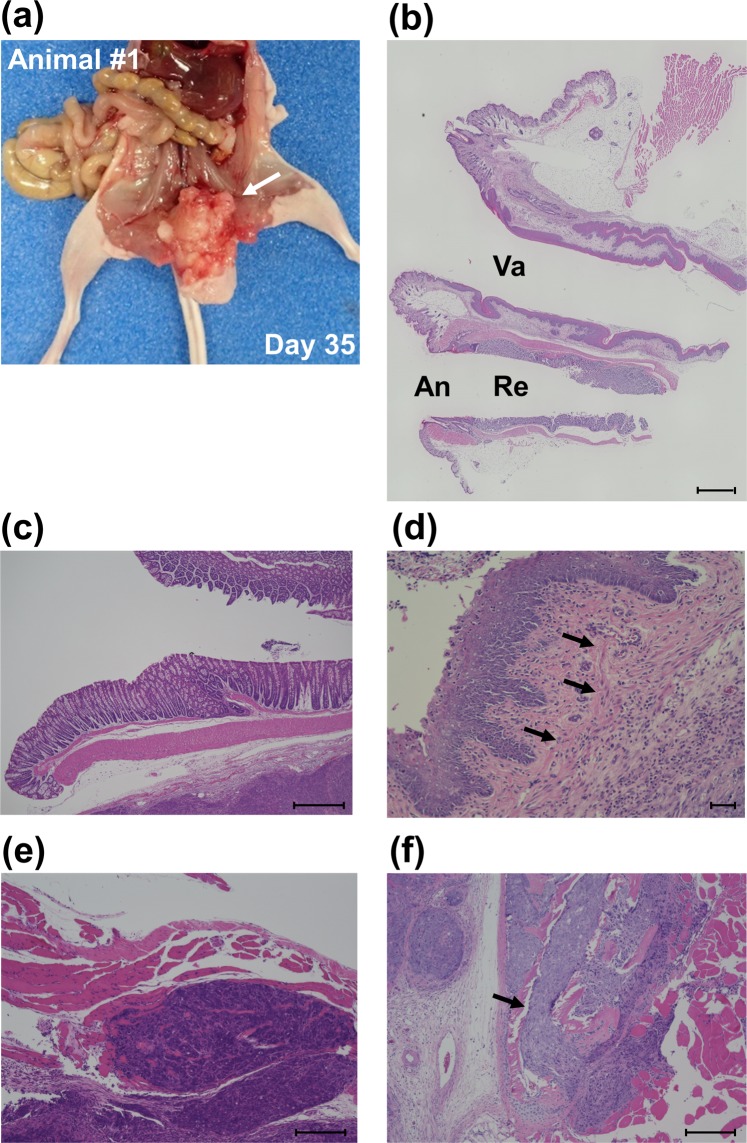
Histological characteristics. (**a**) Representative macroscopic image at necropsy (supine position). IVIS signals indicate the tumor (white arrow). (**b**) A histological image of healthy pelvic organs in a control mouse. Va, vagina; An, anus; Re, rectum. (**c**–**f**) Representative images of H&E stained pelvic organ sections. (**c**) Tumor located in the posterior wall of the rectum (animal #1). Scale bar: 1000 µm. (**d**) Cancer cells involved the muscular layer of the vaginal mucosa (animal #2). Black arrows indicate invasive cancer cells. Scale bar: 50 µm. (**e**) Muscle of the pelvis showing invasive cancer cells (animal #3). Scale bar: 200 µm. (**f**) Cancer cells invading the pubic bone (animal #5). Black arrows indicate invasive cancer cells (n = 5). Scale bar: 200 µm.

Finally, we investigated the pathological validity of our CRC pelvic recurrence model. The macroscopic observation after sacrifice confirmed our imaging findings, as we observed that the injected colon cancer cells grew in the pelvis; there were no metastatic tumors other than in the pelvic space (Fig. 4a). In the five cases of our pelvic recurrence model, there was no involvement of the serosa of the rectal wall (Fig. 4b,c). However, tumor growth in three cases (#2, #3, and #4) extended to the muscular layer of the vaginal mucosa (Fig. 4d). Moreover, tumor invasion into the muscles of the pelvis was observed in all cases (Fig. 4e). It was noteworthy that tumor invasion in two cases (#3, #5) extended to the pubic bone (Fig. 4f).

**Figure 5:**
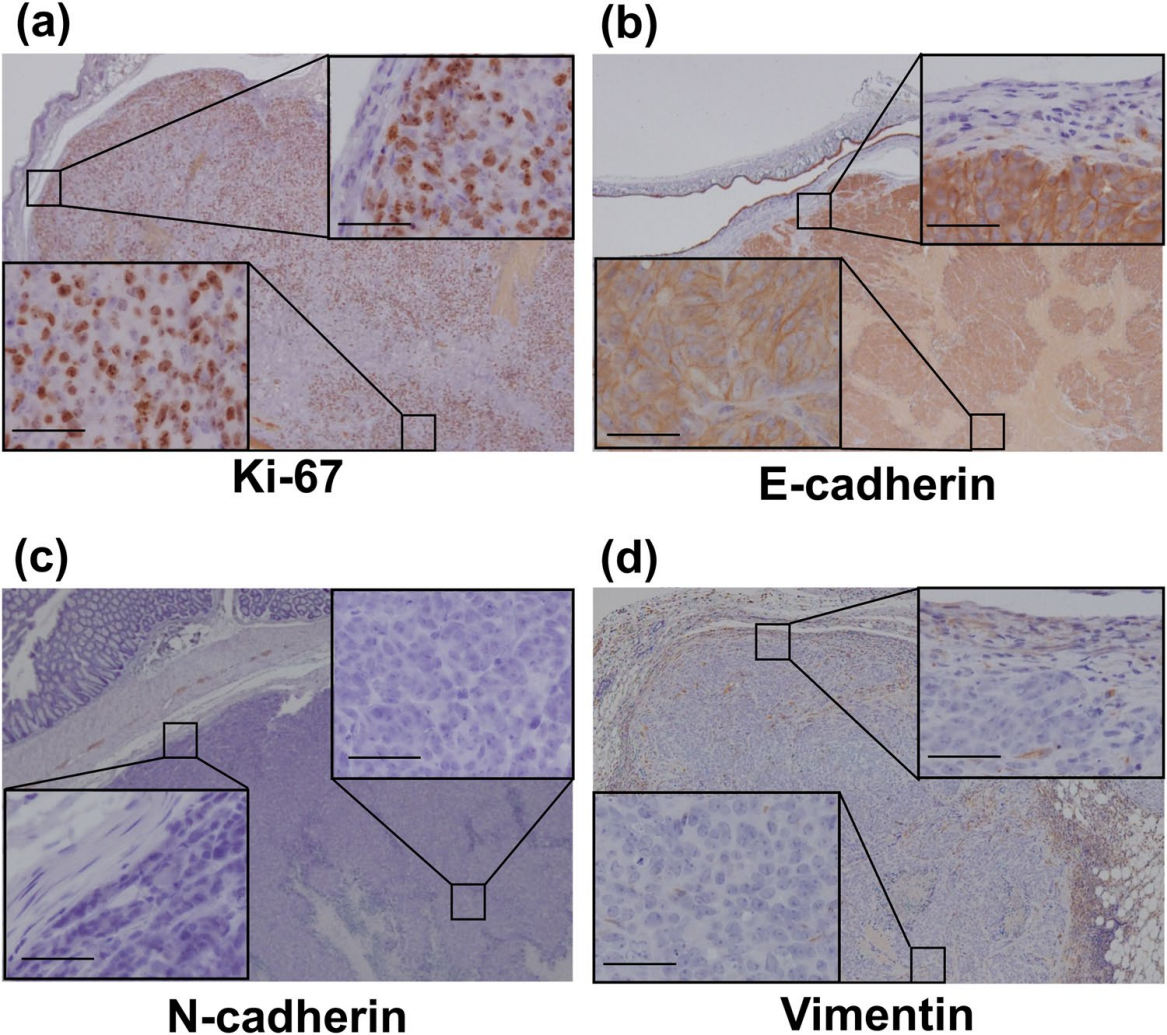
Immunohistochemical characteristics. (**a**) Ki-67: There was no difference in the Ki-67 grade between the invasive edge and the tumor core (+++ vs. +++). (**b**) The expression of E-cadherin was generally positive. (**c**,**d**) The expression of of N-cadherin and vimentin was very weak. There were no significant differences in expression at the tumor core and the invasive edge. Scale bar: 50 µm.

**Figure 6:**
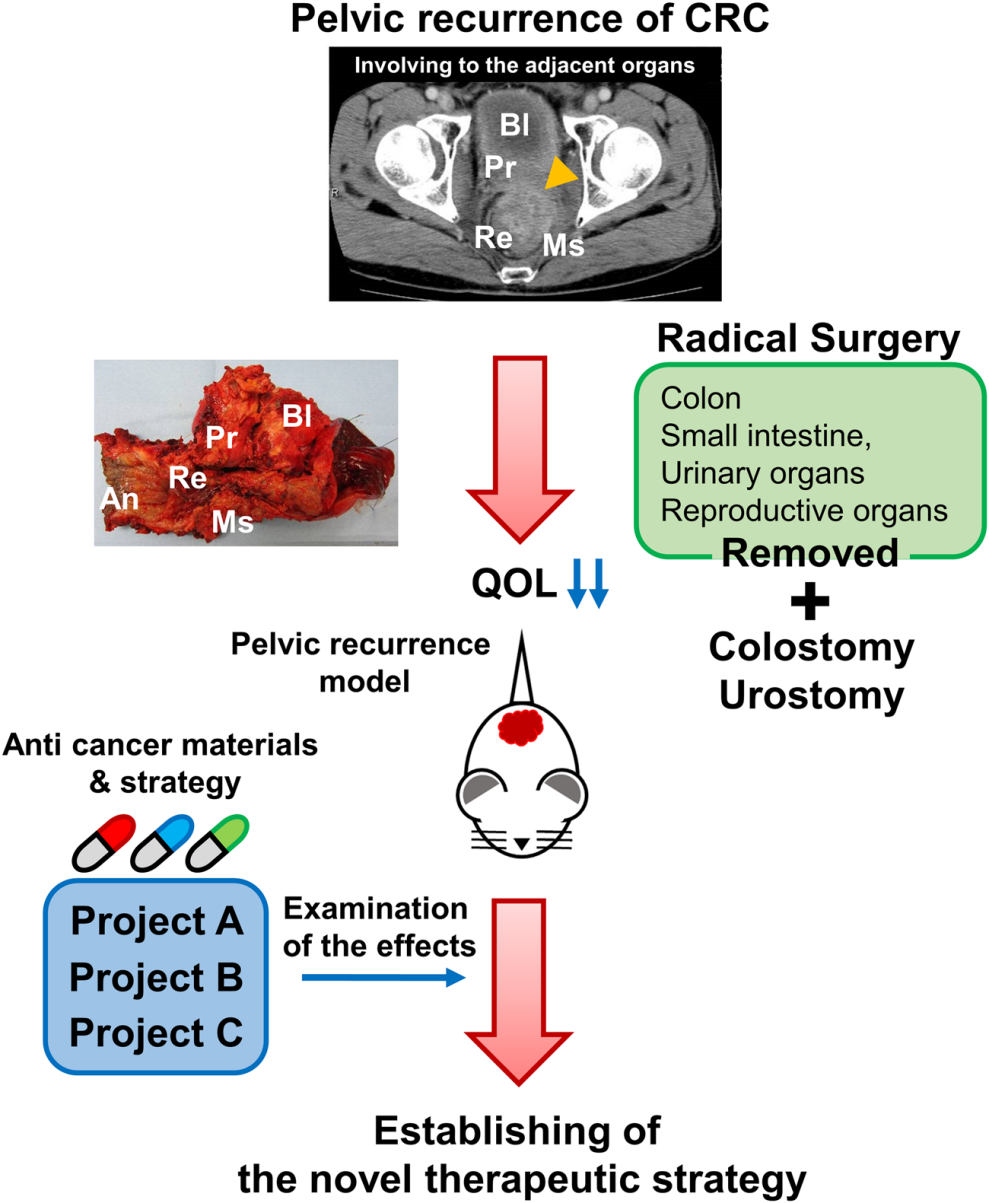
Schematic diagram of the CRC pelvic recurrence model. CT images show the invasion of surrounding structures due to rectal cancer recurrence (yellow arrow). Surgical specimen showing removed pelvic organs. An, anus; Bl, bladder; Ms, levator ani muscle; Pr, prostate; Re, rectum. Several projects have been established using this model.

To investigate the characteristics of our model at the molecular level, the expression of several markers, including markers of epithelial-to-mesenchymal transition (EMT) were examined by immunohistochemistry (IHC). There were no differences in the Ki-67 grade between the tumor core and the invasive edge (+++ vs. +++) (Fig. 5a and Supplementary Fig. S3). E-cadherin was strongly positive in both the tumor core and at the invasive edge (Fig. 5b and Supplementary Fig. S3). In contrast, the expression of N-cadherin and vimentin were generally very weak (Fig. 5c,d, and Supplementary Fig. S3). These results indicate that our model has strong invasive capacity without metastatic ability. These findings suggest that our novel mouse model is useful as a model of CRC local pelvic recurrence (Fig. 6).

## Discussion

Rectal cancer poses a major problem due to the deterioration of QOL caused by local recurrences^11^. Complete resection is considered a standard treatment for this type of tumor^7^, but novel therapeutic strategies are required in order to avoid excessive and invasive surgery. In order to evaluate the effects of new treatments, the degree of imitability of an animal model is very important. Namely, an animal model that has a similar presentation as the human disease should be used. The histopathological assessment of our CRC pelvic recurrence model showed a propensity regarding tumor invasion into the adjacent organs of the pelvis (Fig. 4). Since the detection of tumors within the pelvic space is difficult, we observed engrafted tumors using bioluminescent signals and CT imaging. These imaging modalities were able to assess the tumor volume over time (Fig. 2). DLD-1 cells are non-metastatic and well-differentiated CRC cells. According to the IHC analysis, Ki-67, which reflects cell proliferation, was strongly positive. On the other hand, the expression pattern of EMT markers showed that our model has no metastatic characteristics. Namely, E-cadherin staining was generally a strongly positive reaction and the expression of N-cadherin and Vimentin was very weak (Fig. 5 and Supplementary Fig. S3). We consider this model to be a valid model of localized pelvic CRC recurrence. However, cells with greater metastatic potential might be useful to observe metastatic characteristics; further investigations are needed using highly metastatic cells in this model. In the preliminary test, we had tried to perform some injection methods between the vagina and anus, between the anus and tail horizontally, and within the triangle defined by the vagina, anus, and ischial spine vertically. However, these injection methods did not lead to transplantation into the pelvis. It was the reasons why intraluminal injection, leakage post-injection through the vagina or anus, respectively. The injection site within the triangle defined by the vagina, anus, and ischial spine horizontally led to 100% deposition of the transplanted cells in the pelvis. Using this injection method, it is possible to transplant cells without leakage of the solution through the vagina or anus, due to the wide pelvic space. Firstly, female nude mice were selected to ensure sufficient pelvic space. After that, the growth of injected CRC cells was also observed in male mice (Supplementary Fig. S1). Therefore, our injection method is applicable regardless of gender. However, we could not detect the growth of tumors using a CT scanner in the early stage due to the narrow pelvic space in male mouse. We consider that female mice are more suitable for observation and evaluation than male mice in this model.

During the development of novel therapeutic strategies, the immune system should be considered. Hence, we examined the versatility of our method in wild-type BALB/c mice, which have a functioning immune system. On day 7 after injection, the engrafted tumor could be recognized using bioluminescent signals and CT imaging (Supplementary Fig. S2). These results indicate the reproducibility of our injection method. In consideration of the biological environment in patients, this wild-type model will be more suitable for the evaluation of the effect of novel anti-cancer drugs in the near future.

An endpoint parameter is also a very important factor regarding *in vivo* model development when therapeutic effects are examined. Our CRC pelvic recurrence model had a suitable survival time (Fig. 3). Several CRC animal models, i.e., genetic or surgical models, have been reported and used in various studies^20,21^. The advantage of our model is that, once the injection point was established, the transplant technique was very simple, inexpensive, required little time to establish tumors, and was less invasive compared to other mouse models. Furthermore, the establishment rate of tumors was very high. This is another strength of the model. We believe that this model can be used as a quantifiable and reliable rectal cancer pelvic recurrence model that can be used in studies assessing novel cancer gene therapies, pharmacological studies, and radiotherapy. Several research plans for the establishment of novel therapeutic strategies for rectal cancer pelvic recurrence are underway, employing this model (Fig. 5). Takahara *et al*. demonstrated the effect of boron neutron capture therapy (BNCT) in a prostate cancer model using nude mice^22^; we also considering an evaluation of the effect of BNCT using the mouse model in the current study. Also, we are proceeding with experiments regarding the development of nucleic acid-based drugs, including microRNA therapeutics^23–25^. We are setting up experiments for these projects using this pelvic recurrence model. We hope that other researchers will investigate novel therapeutic strategies for CRC pelvic recurrence using our model.

## Materials and Methods

### Cell lines and cell culture

A human colorectal cancer cell line stably expressing luciferase (DLD-1 clone#1-Luc; JCRB1382) was obtained from the Japanese Cancer Research Resources Bank (JCRB, Osaka, Japan). DLD-1 clone#1-Luc cells were cultured in RPMI-1640 (Hyclone, Logan, UT, USA) supplemented with 10% (v/v) heat-inactivated fetal bovine serum, in a 5% CO_2_ atmosphere at 37 °C.

### Animals

Six- to eight-week-old female BALB/c nude mice were purchased from Japan SLC, Inc., (Hamamatsu, Japan). The mice were housed no more than 5 mice per plastic cage on wood chip bedding with free access to water and food and maintained under conditions of controlled temperature (21 ± 2 °C), humidity (50 ± 10%), and lighting (12 h-12 h light-dark cycle). All mice were held for a one-week acclimatization period prior to test initiation. All manipulations and treatments of the mice were performed in accordance with procedures outlined in the Guide for the Care and Use of Laboratory Animals of the National Institutes of Health^26^. The protocol was approved by the Osaka Medical College Animal Care and Use Committee (approval number 28109). At the termination of the study, all mice were euthanized under isoflurane anesthesia.

### Transplantation method

While under isoflurane inhalation using a laboratory animal anesthesia system (Shinanoseisakusho CO., LTD, Tokyo, Japan), 6 × 106 DLD-1 clone#1-Luc cells in 0.15 ml of phosphate-buffered saline (PBS) were inoculated horizontally into the pelvic cavity of 5 BALB/c nude mice using a 27 G needle. The puncture site was located within the triangle defined by the vagina, anus, and ischial spine) (Fig. 1). The needle insertion depth ranged from 0.5 cm to 1.0 cm.

### Bioluminescence imaging

Under the same sedation conditions, the mice were injected intraperitoneally with D-luciferin potassium salt (Wako Pure Chemical Corporation, Osaka, Japan) at 3 mg/mouse. Bioluminescent signals received during the 6 min acquisition time were quantified using the IVIS Lumina *in vivo* imaging system and Living Image Software Version 4.0 (Perkin Elmer, Waltham, MA, USA). Tumor area was measured using the region of interest (ROI) contour tool.

### CT scanning

Under the same sedation conditions, the tumor volume in each mouse was calculated using a Latheta CT scanner (LCT-200 series, Hitachi, Tokyo, Japan) without a contrast agent. Tumor area was measured using the ROI contour tool. The scanned images were reconstructed in three dimensions (3D) and analyzed using VG Studio max 2.2 (Volume Graphics GmbH, Charlotte, NC, USA). This CT system provides a tube power voltage of 50 kV and a tube current of 0.5 mA. The average exposure time was 11 min for an average of 100 scans. The pixel size was 1024 × 1024. The voxel size was 48 × 192 μm. The images were reconstructed using traditional filter functions.

### Histopathological assessment

At necropsy, all specimens were routinely removed by total pelvic exenteration, and were fixed in 10% formaldehyde solution in phosphate buffer. Then, the specimens were processed through paraffin embedding (Nara-byouri Laboratory Co., Ltd., Nara, Japan). All tissues were cut into sequential 4 µm thick sections and stained with hematoxylin and eosin (Muto Pure Chemical Co., Ltd., Tokyo, Japan) for the histopathological examination.

### Immunohistochemical assessment

Immunohistochemistry was performed on 5 µm-thick formalin-fixed, paraffin-embedded tissue sections mounted on adhesive glass slides. The sections were deparaffinized with xylene and hydrated in an ethanol series. Then, the specimens were pretreated with pH 7.4 bovine serum albumin in (PBS), and endogenous peroxidases were blocked by incubating the sections with 3% hydrogen peroxide in distilled water. The tissue specimens were incubated with antibodies recognizing Ki-67 (Agilent Technologies, Inc., Santa Clara, CA, USA 1:100), E-cadherin (Cell Signaling Technology, Inc., Danvers, MA, USA, 1:100), N-cadherin (Cell Signaling Technology, Inc., Danvers, MA, USA, 1:100), and vimentin (Cell Signaling Technology, Inc., Danvers, MA, USA, 1:200) overnight at room temperature. The, the tissue sections were treated with the secondary antibody (Simple Stain MAX-PO (M) kit, Nichirei, Inc. Tokyo, Japan). The specimens were inspected with a microscope (Eclipse E600, Nikon, Japan) and photographed. For Ki-67 staining results, “+” indicates that the number of positive cells in the tissue in a microscope field was less than 20, “++” indicates that the number of positive cells was 20–50, “+++” indicates that the number of positive cells was 50–100, and “++++” indicates that positive cells were distributed throughout the field.

### Statistical analysis

Statistical analysis was performed using JMP 14 for Windows (SAS Institute Inc., Cary, NC, USA). Student’s t-test, the Mann-Whitney *U* test, and the χ^2^ test were used to compare continuous and categorical variables, respectively, with two-sided *p* < 0.05 indicating significance. Survival analysis was performed using Kaplan-Meier survival curves with log-rank statistics.

## Supplementary information

Supplementary Information
