# Supplementary material for: An In Vivo Mouse Model of Pelvic Recurrence of Human Colorectal Cancer
Source: Sci Rep. 2019 Dec 23;9:19630. doi: 10.1038/s41598-019-56152-0 (PMC6928073; doi:10.1038/s41598-019-56152-0)
Supplement: Supplementary file 1 — Supplementary Information [file 41598_2019_56152_MOESM1_ESM.docx]

**An *In Vivo* Mouse Model of Pelvic Recurrence of Human Colorectal Cancer**

Masashi Yamamoto^1)*^, Kohei Taniguchi^1), 2) ϯ^, Shinsuke Masubuchi^1)^, Tomo Tominaga^1)^, Yosuke Inomata^1)^, Akiko Miyamoto^1)^, Taka-Aki Ishizuka^3)^, Takashi Murakami^4)^, Wataru Osumi^1)^, Hiroki Hamamoto^1)^, Keitaro Tanaka^1)^, Junji Okuda^1)^, Kazuhisa Uchiyama^1)^

Departments of General and Gastroenterological Surgery, Osaka Medical College, Osaka, Japan^1)^

Translational Research Program, Osaka Medical College, Osaka, Japan ^2)^

Division of Research Equipment and Device, Osaka Medical College, Osaka, Japan ^3)^

Faculty of Medicine, Saitama Medical University, Saitama, Japan^4)^

**^*^ Correspondence:** Masashi Yamamoto, MD, Ph.D.

Department of General and Gastroenterological Surgery, Osaka Medical College

2-7 Daigakumachi, Takatsuki, Osaka 569-8686, Japan

Tel: +81-72-683-1221; Fax: +81-72-684-6541

E-mail: [sur138@osaka-med.ac.jp](mailto:sur138@osaka-med.ac.jp)

**^Ϯ^ Co-Correspondence:** Kohei Taniguchi, MD, Ph.D.

Translational Research Program, Osaka Medical College, Osaka, Japan

2-7 Daigakumachi, Takatsuki, Osaka 569-8686, Japan

Tel: +81-72-684-6874; Fax: +81-72-684-6525

E-mail: [sur144@osaka-med.ac.jp](mailto:sur144@osaka-med.ac.jp)

**Supplementary Figure S1. Representative macroscopic image at necropsy of a male BALB/c nude mouse (supine position)**

**
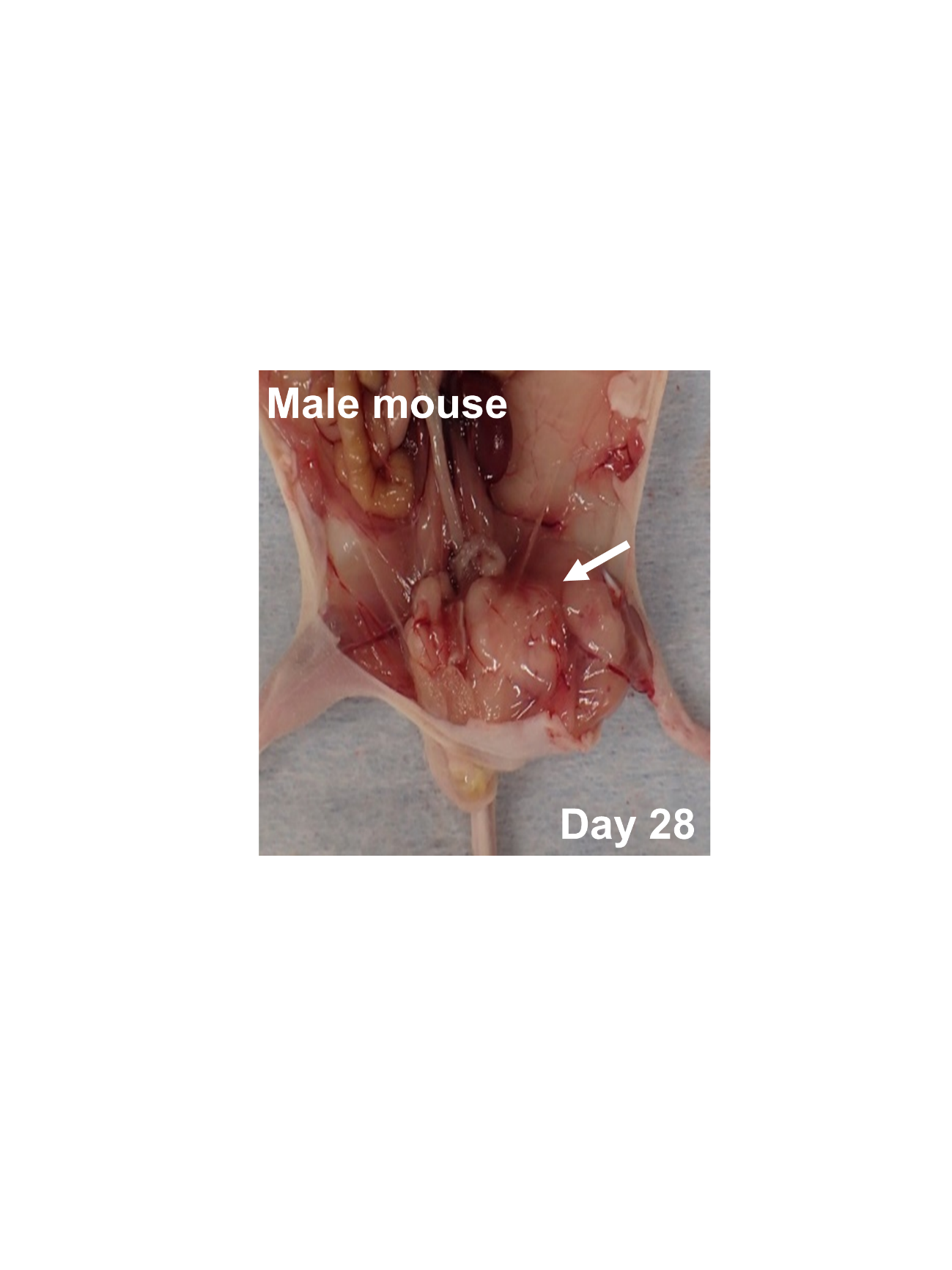
**

**Supplementary Figure S2. Representative images of tumor growth in the CRC pelvic recurrence model (BALB/c mice and colon26-Luc)**

**
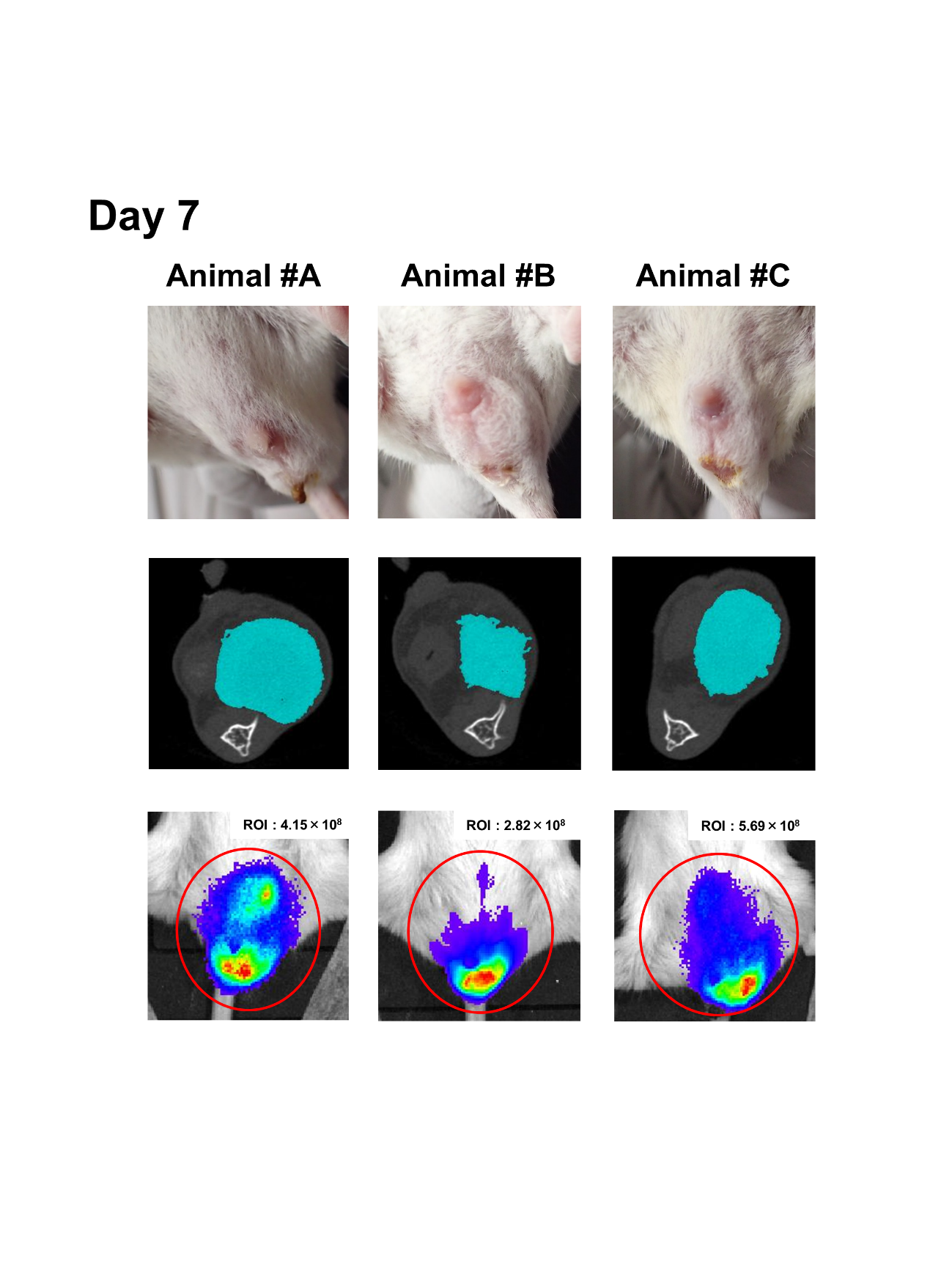
**

**Supplementary Figure S3.** **Immunohistochemical staining of Ki-67, E-cadherin, N-cadherin, and vimentin**

**
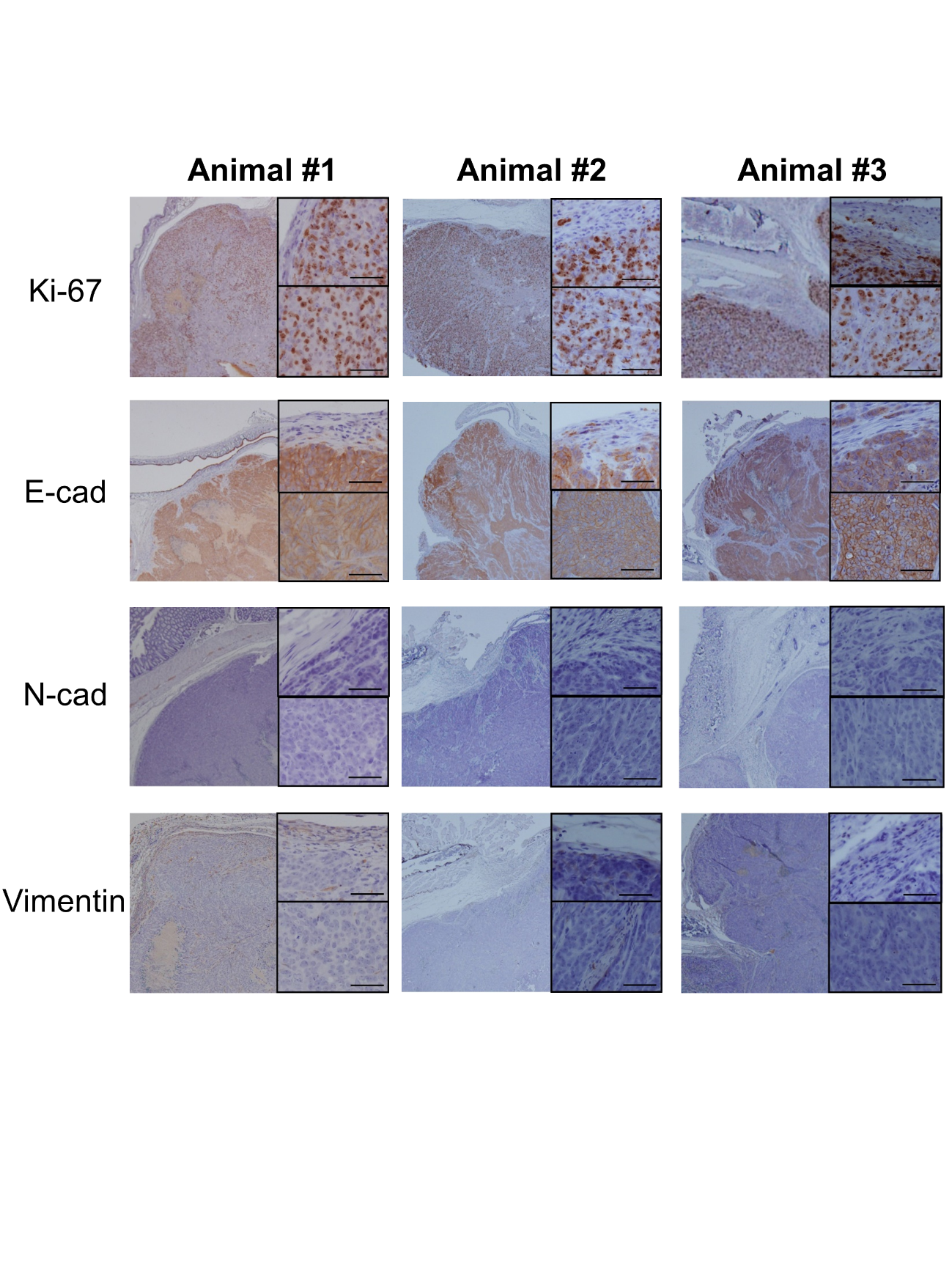
**

**SUPPLEMENTARY FIGURE LEGENDS**

**Supplementary Figure S1.** Pelvic metastasis was confirmed by necropsy in male mice using same injection method.

**Supplementary Figure S2.** On day 7 after injection, the engrafted tumor could be recognized using bioluminescent signals and CT imaging.

**Supplementary Figure S3.** Immunohistochemical staining of Ki-67, E-cadherin, N-cadherin, and vimentin in three mice. Protein expression was evaluated at the tumor core and invasive edge. Scale bar: 50 µm.
